# Validation and comparison study of three urbanicity scales in a Thailand context

**DOI:** 10.1186/s12889-016-2704-y

**Published:** 2016-01-14

**Authors:** Wiroj Jiamjarasrangsi, Wichai Aekplakorn, Thosporn Vimolkej

**Affiliations:** 1 Department of Preventive and Social Medicine, Faculty of Medicine, Chulalongkorn University, Rama IV Road, Pathumwan, Bangkok, 10330 Thailand; 2 Department of Preventive and Social Medicine, King Chulalongkorn Memorial Hospital, Thai Red Cross, Bangkok, 10330 Thailand; 3 Department of Community Medicine, Faculty of Medicine, Ramathibodi Hospital, Mahidol University, Bangkok, 10400 Thailand

**Keywords:** Urbanicity, Scale, Validity, Reliability

## Abstract

**Background:**

Validity and reliability of an urbanicity scale is of utmost importance in developing effective strategies to minimize adverse social and health consequences of increased urbanization. A number of urbanicity scales for the quantitative assessment of the “static” feature of an urban environment has been invented and validated by the original developers. However, their comparability and robustness when utilized in another study context were not verified. This study aimed to examine the comparability, validity, and reliability of three urbanicity scales proposed by Dahly and Adair, Jones-Smith and Popkin, and Novak et al. in a Thailand context.

**Methods:**

Urban characteristics data for 537 communities throughout Thailand were obtained from authoritative sources, and urbinicity scores were calculated according to the original developers’ algorithms with some modifications to accommodate local available data. Comparability, dimensionality, internal consistency, and criterion-related and construct validities of the scores were then determined.

**Results:**

All three scales were highly correlated, but Dahly and Adair’s and Jones-Smith and Popkin’s were more comparable. Only Dahly and Adair’s scale achieved the unidimensionality assumption. Internal consistency ranged from very poor to high, based on their Chonbach’s alpha and the corrected item-scale correlation coefficients. All three scales had good criterion-related validity (when compared against the official urban–rural dichotomy and four-category urbanicity classification) and construct validity (in terms of their relation to the mean per capita monthly income and body mass index).

**Conclusions:**

This study’s results ensure the utility of these three urbanicity scales as valid instruments for examining the social and health impacts of urbanicity/urbanization, but caution must be applied with comparisons of urbanicity levels across different studies when different urbanicity scales are applied.

**Electronic supplementary material:**

The online version of this article (doi:10.1186/s12889-016-2704-y) contains supplementary material, which is available to authorized users.

## Background

Since the proportion and number of population residing in urban areas are increasing worldwide [1], influence of urban features on human health has greater importance [2]. This is especially true for developing countries where the urbanization rate is higher but relevant data is insufficient [3]. Impact of urbanicity on health is complex and can be both positive and negative [4]. Sufficiently detailed evidence is therefore needed for proper public health planning to maximize benefits while simultaneously minimizing the detrimental impacts of urbanization on residents’ health [5]. Although evidence is available on the association of urbanicity and urbanization with human health in developing countries, most of the studies have relied on the urban–rural dichotomy in the exposure assessment. This procedure of urban exposure assessment is inadequate since it is not supportive for a detailed investigation of the nuance pattern of urbanization and health association [6, 7].

A number of researchers have developed urbanicity scales for quantitative assessment of the “static” feature of urbanization [7–16]. These scales have enabled the investigation of delicate patterns of urbanization and urbanicity impacts on population health [17]. An example is the study by Gordon-Larsen et. al, in which the urbanicity scale was able to demonstrate delicate patterns of simultaneous impacts of urbanicity and urbanization on adult overweightness in China during 1991-2009 [18]. Another example is Riha et al’s study in which the urbanicity scale developed by Novak et al. was used to reveal that even small increases in urbanicity in rural areas was associated with higher prevalence of chronic disease risk factors in Subsaharan Africa [19]. However, the majority of the studies did not report the properties of the urbanicity scales [17].

Some of these scales have been formally validated, including those developed by Dahly and Adair in the Phillippines context [7], Van de Poel et al. and Jones-Smith and Popkin in a China context [10, 14], and Novak et al. in a multi-country context (Ethiopia India Peru) [15]. Van de Poel et al’s score is derived from the factor analysis of a set of 26 community level characteristics that reflect a community’s level of urbanicity [10]. The scoring system of the other three scales is based on the equal-weight 10-point score for each component, with the number of components being 7 for Dahly and Adair’s [7] and Novak et al’s [15] and 12 for Jones-Smith and Popkin’s scales [14]. The authors reported that validity of these scales was satisfactory in terms of unidimensionality, internal consistency, temporal stability, criterion–related validity when compared to both the official urban–rural dichotomy and four-category urban classification, and construct validity for various health and social outcomes. However, their robustness when utilized in other study settings is unknown. In addition, since these scales used different indicators, or variables, in their development (probably depending on local availability of related data), their comparability is still unknown [17]. These deficiencies limit international comparison and generalization of study findings about urbanization, urbanicity, and health.

In this study, the potential utility of the previously validated urbanicity scales was further investigated, especially those based on the equal-weight 10-point score for each component. Specifically, this study’s objectives were: (a) to examine the comparability of the urban scales proposed by Dahly and Adair [7], Jones-Smith and Popkin [14], and Novak et al. [15] in classifying the urbanicity level of villages and communities in Thailand; (b) to evaluate the validity and reliability of these scales in the Thailand context.

## Methods

### Study area and data sources

Thailand is a middle-income country located in Southeast Asia. Its administrative structure is divided into 77 provinces. Each province is divided into districts, and the districts are further divided into sub-districts. Each sub-district is further divided into villages (out of municipal areas) or communities (in municipal areas). The study samples were 537 villages and communities multi-stage randomly selected, and their residents were utilized in the 4^th^ national health examination survey of Thailand conducted in 2008-2009 [20]. These villages and communities were located in 17 provinces, 90 districts, and 404 sub-districts/municipalities throughout the country. Their average area was 2.864 km^2^, which is comparable to the approximate size of 2 km^2^ of the Philippines’ barangay) [7]. The study was approved by the Institutional Review Board of the Faculty of Medicine, Chulalongkorn University, Bangkok, Thailand (Certificate of Approval: COA No. 754/2014, IRB No. 324/57, Date of Approval: November 6, 2014).

Village and community level variables utilized in the composition of urbanicity scales were obtained mostly from the Fundamental Database at Village Level (Gor Chor Chor 2 Kor) [21]. This database was developed and is maintained by the Department of Community Development at the Ministry of Interior of Thailand. Data of 33 indicators on seven socio-economic aspects at village level throughout the country were updated biannually, and those for 2007 were utilized in this study. However, data for 166 communities in the municipal areas were mostly unavailable in this database. Data from the densest village in the same or adjacent sub-district were utilized to represent the data for the particular communities in their municipal areas. In addition, variables which were not available in this database were further obtained from relevant government sources both by request and via the Internet (including: the proportions of households with flush toilet, color television, cable TV, and gas cooker; local availability of vocational schools and colleges, sewage treatment system, a movie theatre, bus and train stations, fresh market, supermarket, and healthcare facilities; and per capita monthly income) [22–33]. The majority of the variables were village/community level data. The exception were the data about the local availability of a movie theatre, bus station, fresh market and supermarket, which were at sub-district level; and the data about the proportions of households with flush toilet, color television, cable TV, and gas cooker were at the district level.

Proportions of missing data ranged between 0 to 5% for each variable and one percent on average, with the exception only for educational variables of which missing data were up to13.8%. They were replaced by random hot-deck imputation within class (sub-district and municipal-non municipal areas) to facilitate statistical analysis [34].

### Urbanicity scales

Three previously validated urbanicity scales which were proposed by Dahly and Adair [7], Novak et al. [35], and Jones-Smith and Popkin [14] based on the equal-weight 10-point score for each component were utilized in this study. The components used in each scale scoring system were: seven for Dahly and Adair’s (population size, population density, communications, transportation, educational facilities, health services, and markets; the total scale ranges from 0 to 70); seven for Novak et al’s (population size, economic activity, built environment, communications, education, health services, and diversity, which comprise two separate scores related to variance in housing quality and variance in the number of years mother has spent in education; the possible score ranges from 0 to 70); and twelve for Jones-Smith and Popkin’s (population density, economic activity, traditional markets, modern markets, transportation infrastructure, sanitation, communications, housing, education, health infrastructure, social services, and diversity in variation in community education level and community income level; possible score ranges from 0 to 120). However, some modifications on the variables utilized in the scale scoring procedure were made to suit the availability of the existing local database in Thailand. Details of these modifications are presented as follows, while more specific details for all variables as well as scoring procedures and data sources are provided in the Additional file 1.

Dahly and Adair’s scale: In the communications component, the item about newspaper service was omitted, and the remaining total score of 8 was adjusted to 10. In the education component, the item about complete school was dropped, while the item about nursery/preschool was added. In the transportation component, the item of “the presence and availability of both bus and jeepney (taxi) service” was modified to include motorcycle service. In the health service component, medical, physiotherapy, nurse and midwife, dental clinics, and medical technician clinics were used in place of Dahly and Adair’s maternal health clinics, family planning clinics, puericulture centers, and rural health units. In the market component, the availability of a fresh market was used in place of grocery store, and small grocery store was used in place of “sari-sari” store; the drug store item was omitted, and the remaining score was adjusted to 10.

Novak et al’s scale: In the education component, adult female’s education was used instead of mother’s education. Variance in per capita monthly income was used in place of variance in housing quality in the diversity component.

Jones-Smith and Popkin’s scale: The traditional market component was based solely on the availability of a fresh market within or nearby the village. The transportation component was based on the availability of long distance bus and train stations/terminals. The sanitation component was based solely on the availability of a well-equipped sewage treatment system [28], not just having storm drains. In the communications component, the item about newspaper was omitted, and the remaining score was adjusted to 10. In the social service component, the availability of a community vocational training center in the community and an assistance center for various kinds of disabled persons in the community or nearby were used in place of insurance for women and children [35]. Since free medical insurance has been universal in Thailand since 2002, a score of 2.5 was assigned for all studied villages and communities [36].

The scale proposed by Van de Poel et al. [10] for which the weight of each component was based on factor analysis result was not included in this study since there were no detailed data to utilize in the scoring procedure.

### Statistical analysis

#### Comparison among the urbanicity scales

To facilitate the comparison among the three urbanicity scales, the scores were standardized into the same maximum score of 100. Pattern of correlations among the three standardized scales were examined by the scatter plot matrix, and the corresponding Pearson’s correlation coefficients were determined. The coefficient values of < 0.50 indicated low correlation, 0.50-0.75 high correlation, and >0.75 very high correlation [37, 38]. In addition, paired *t*-test was conducted to determine the magnitudes of difference among the three standardized scales [39]. Standardized scores were also utilized in the examination of criterion-related and construct validity to facilitate comparison across the three urbanicity scales.

#### Scale properties and reliability

In determining dimensionality, or whether the various components of the scale actually measure a single construct (in this case, urbanicity), exploratory factor analysis was conducted without restricting the number of factors estimated [40]. The dimensionality of the scales was assessed by the number of factors with eigenvalues >1 and a scree plot [41].

The internal consistency of the scale, or degree of interrelatedness of the components within the scale, was examined by using Cronbach’s alpha, with corrected item-scale correlations also reported [40, 41]. An alpha of 0.60-0.70 indicated an acceptable level of reliability and 0.8 or greater a very good level, and corrected item total correlations of r ≥ 0.30 indicated that the subscale items were well-correlated with remaining components in the scale.

#### Criterion-related and construct validities

Criterion-related validity of the scales can be assessed by comparing the degree of agreement between the scales and the standard measurement [40]. Since there is still no standard measurement in the case of urbanicity, the scales were compared to the existing official classification of urban–rural dichotomous classification as well as a four-category classification: city municipality, town municipality, sub-district municipality, and sub-district administrative organization jurisdiction (rural area). In comparing the scales to the dichotomous urban/rural classification, the scales were dichotomized (based on the receiver operator characteristic curve determination of maximized sensitivity and specificity) into high- and low-urbanicity and then compared with the resulting categories using the kappa statistic for agreement beyond chance [14]. A Kappa statistic of 1 would indicate perfect agreement, over 0.80 excellent, 0.61-0.80 good, 0.41-0.60 moderate, and 0.21-0.40 fair agreement [39]. Criterion-related validity of the scales against the four category classifications was then conducted by using Spearman’s rank correlation, with the coefficient value of > 0.75 considered as very strong, 0.50-0.75 as strong, and <0.50 as weak correlation [37].

#### Construct validity

To evaluate the construct validity, or the extent to which the scales coincided with the phenomena known to differ by urban status [40], the relationship of urbanicity scales with per capita monthly income and body mass index was determined by regression analysis. Per capita income and body mass index had been previously proved to vary according to urbanicity level [8, 10, 14]. Data about per capita monthly income (at sub-district level) was obtained from the Thailand National Statistical Office [42], while the data about body mass index was obtained from the 4^th^ National Health Examination Survey database, which contains 17,275 survey subjects throughout Thailand except Bangkok) [20]. The difference of per capita monthly income and body mass index according to quintile of urbanicity scores was then assessed by analysis of variance (ANOVA) with Bonferoni correction for multiple-comparison tests [39]. The p-value of <0.05 was considered as statistically significant. In addition, Spearman’s rank correlation of the quintile of urbanicity scales with per capita monthly income and body mass index was also determined.

## Results

### Comparison among the urbanicity scales

Of all 15 urbanicity components, three were shared by all three urbanicity scales (including communications, education, and health components), while the other five components were utilized by two scales. The built environment component of Novak et al. was also quite comparable to the combined housing, sanitation, and transportation components of the Jones-Smith and Popkin’s scale. Actual scores of these components across the three scales were however different due to their different scoring algorithms (Table 1).

**Table 1:** Urbanicity scores of the three urbanicity scales

Component	Novak et al.					Dahly and Adair					Jones-Smith and Popkin				
	Mean	(SD)	Min	-	Max	Mean	(SD)	Min	-	Max	Mean	(SD)	Min	-	Max
Population size	1.62	(0.75)	1.00	-	6.00	1.62	(0.75)	1.00	-	6.00					
Population density						1.39	(0.97)	1.00	-	7.00	4.34	(1.08)	0.00	-	7.50
Economic activity	4.83	(3.93)	0.00	-	10.00						2.67	(2.34)	0.00	-	10.00
Built environment	8.07	(0.84)	2.00	-	10.00										
Housing											8.62	(1.06)	5.20	-	10.00
Sanitation											1.08	(3.11)	0.00	-	10.00
Transportation						6.51	(2.37)	0.00	-	10.00	3.84	(1.45)	1.67	-	10.00
Communication	6.30	(1.65)	1.80	-	9.90	7.04	(1.94)	1.27	-	9.27	5.95	(1.14)	2.00	-	9.10
Education	4.04	(2.03)	1.00	-	9.40	2.86	(2.11)	0.00	-	10.00	3.34	(0.99)	1.62	-	7.31
Health	3.69	(2.15)	2.00	-	10.00	2.88	(2.49)	0.00	-	10.00	2.79	(2.33)	0.00	-	10.00
Markets						3.24	(1.55)	0.00	-	6.67					
Traditional market											3.69	(4.83)	0.00	-	10.00
Modern market											0.37	(0.74)	0.00	-	4.50
Social services											4.44	(1.66)	2.50	-	10.00
Diversity	5.49	(2.25)	1.00	-	10.00						4.93	(1.31)	2.00	-	10.00
Summary score	34.03	(7.03)	16.20	-	58.50	25.54	(6.25)	9.53	-	53.48	46.05	(12.25)	23.57	-	85.45
Standardized score	48.61	(10.05)	23.14	-	83.57	36.48	(8.94)	13.61	-	76.40	38.38	(10.21)	19.64	-	71.21

Min=minimum

Max=Maximum

SD=Standard deviation

Based on the standardized values, linear correlations among the three scores were high, especially between Dahly and Adair’s and Jones-Smith and Popkin’s (Table 2). The standardized urbanicity score assessed by Novak et al’s instrument was much higher, while those scores assessed by Dahly and Adair’s and Jones-Smith and Popkin’s instruments were quite comparable, although their difference was still statistically significant (Table 2).

**Table 2:** Comparison among the three standardize urbanicity scores

Parameter	Mean	(SD)	(95 % confidence interval)		Pearson’s correlation coefficient^a^
Score					
Novak et al.	48.61	(10.05)	(47.76,	49.46)	
Dahly and Adair	36.48	(8.94)	(35.72,	37.24)	
Jones-Smith and Popkin	38.38	(10.21)	(37.51,	39.24)	
Difference					
Novak et al. – Dahly and Adair	12.13	(7.91)	(11.46,	12.80)	0.66
Novak et al. - Jones-Smith and Popkin	10.23	(7.82)	(9.57,	10.89)	0.70
Dahly and Adair - Jones-Smith and Popkin	−1.90	(7.27)	(−2.51,	−1.28)	0.72

^*a^ =all with p < 0.001

SD=Standard deviation

#### Scale properties and reliability

Based on the number of factors with eigenvalues >1, factor analysis results showed that only Dahly and Adair ‘s urbanicity scale achieved unidimentionality (Table 3). For Novak et al’s scale, no factor had eigenvalues >1, and the dimensions that were not well co-varied with the main factor were population size and educational facility components. For the Jones-Smith and Popkin’s scale, two factors emerged with eigenvalues > 1, and the dimensions that were not well co-varied with the main factor were the economic, educational, housing quality, and diversity components (details not shown).

**Table 3:** Dimensionality, reliability, and criterion-related validity of three urbanicity scales

Test parameter		Novak et al.		Dahly and Adair		Jones-Smith and Popkin	
		Present^a^	Previous^b^	Present^a^	Previous^b^	Present^a^	Previous^b^
Dimensionality							
	Number of factor(s)	0	1	1	n/a	2	1
Internal consistency							
	Chonbach’s alpha	0.48	n/a	0.51	0.87	0.74	0.89
	Item scale correlations						
	Population size	0.44	0.50	0.46	0.72		
	Population density			0.45	0.64	0.71	0.47
	Economic activity	0.44	0.88			0.72	0.70
	Built environment	0.40	0.73				
	Housing					0.76	0.80
	Sanitation					0.70	0.77
	Communication	0.44	0.80	0.52	0.70	0.72	0.68
	Transportation			0.49	0.56	0.72	0.40
	Education	0.52	0.85	0.54	0.48	0.71	0.67
	Health	0.37	0.62	0.41	0.75	0.69	0.62
	Markets			0.42	0.80		
	Traditional market					0.72	0.61
	Modern market					0.73	0.75
	Social services					0.75	0.51
	Diversity	0.45	0.40			0.72	0.67
Criterion-related validity							
*Compared with urban–rural dichotomy classification*							
	Observed Agreement	64.43 %	88.10 %	71.88 %	n/a	74.86 %	74 %
	Expected Agreement	52.20 %	49.80 %	51.47 %	n/a	50.48 %	51 %
	Kappa Statistic	0.26	0.76	0.42	n/a	0.49	0.48
	*p-value*	*<0.001*	*<0.0001*	*<0.001*	*n/a*	*<0.001*	*<0.05*
*Compare with four-category urban classification*							
	Spearman’s Correlation	0.37	0.84	0.45	n/a	0.58	0.75-0.78
	*p-value*	*<0.001*	*<0.0001*	*<0.001*		*<0.001*	

^a^Present validation results

^b^Previous validation results by the original scale developers

n/a=not available

The Chonbach’s alpha and corrected item-scale correlation coefficients of the Jones-Smith and Popkin’s scale were quite high (0.69-0.76), indicating good internal consistency for overall scale and each component. On the other hand, those for Novak et al’s and Dahly and Adair ‘s urbanicity scales were poor (<0.60) or very poor (<0.50). These magnitudes of internal consistency in this study were lower than previous validation results, especially for those of Novak et al’s and Dahly and Adair ‘s urbanicity scales (Table 3).

### Criterion-related validity

A comparison with the official urban–rural dichotomous classification showed that the criterion-related validity was moderate for Jones-Smith and Popkin’s and Dahly and Adair ‘s scales, as shown by their kappa values of >0.40 (Table 3). The kappa of Jones-Smith and Popkin’s scale was the highest (0.49), while that of Novak et al’s scale was poor and lowest (0.26). Similarly, when the urbanicity scores were compared with the official four-category urban classification, the criterion-related validity was high for Jones-Smith and Popkin’s (Spearman’s rank correlations = 0.58, *p* < 0.001), poor for Dahly and Adair’s and Novak et al’s scales (Spearman’s rank correlations = 0.45 and 0.37 respectively, *p* < 0.001).

This study’s criterion-related validity for the Jones-Smith and Popkin’s scale was comparable to the previous validation result for the official urban–rural dichotomy classification (kappa = 0.49 versus 0.48), but much lower for the official four-category urban classification (Spearman’s rank = 0.58 versus 0.76). Concerning Novak et al’s scale, this study’s criterion-related validity was much lower than the previous validation result for the dichotomous urban–rural classification (kappa = 0.26 versus 0.76) (Table 3).

**Fig. 1:**
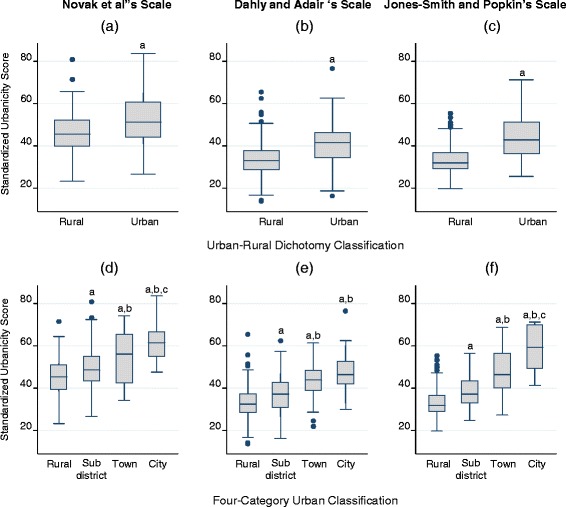
Distribution of the standardized urbanicity scores by two official rural–urban classifications: (**a**) and (**d**) for Novak et al’s scale; (**b**) and (**e**) for Dahly and Adair’s scale; (**c**) and (**f**) for Jones-Smith and Popkin’s scale. **a** = different from “rural” at *p* < 0.05, **b** = different from “sub-district” at *p* < 0.05, **c** = different from “town” at *p* < 0.05

However, when examining the mean scores of these scales according to the official four-category urban classification, results showed that the difference in mean scores by formal urban classification were highly significant for all three scales (Fig. 1). The exception was the non-significant different means score between the “town” and “city” of Dahly and Adair’s scale (Fig. 1d).

### Construct validity

**Fig. 2:**
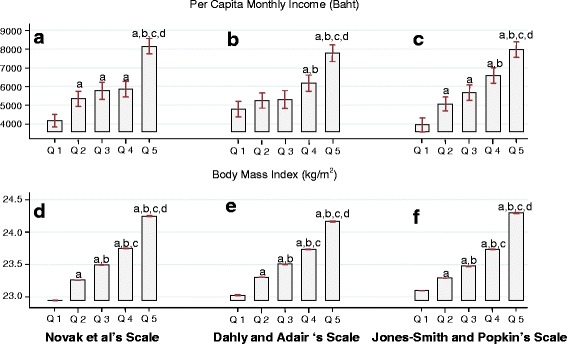
Distribution of per capita monthly income and body mass index by quintiles of urbanicity level: (**a**) and (**d**) for Novak et al’s scale; (**b**) and (**e**) for Dahly and Adair’s scale; (**c**) and (**f**) for Jones-Smith and Popkin’s scale. Box represents mean, bar represents the 95 % confidence interval. **a** = different from Quintile1 at *p* < 0.05, **b** = different from Quintile 2 at *p* < 0.05, **c** = different from Quintile3 at *p* < 0.05, **d** = different from Quintile 4 at *p* < 0.05, Q = quintile of urbanicity level

Construct validity of the three scales were assessed by regression analyses to determine the association of the urbanicity score with per capita monthly income and body mass index of the population according to quintile of urbanicity scales. Results showed that the associations were significant and comparable for all three scales (Table 4). One unit increase in the standardized urbanicity scores was associated with a 110–136 baht increase in per capita monthly income and 0.42-0.45 kg/m^2^ increase in body mass index. Examination of construct validity of the urbanicity scores by plotting the means per capita monthly income and body mass index against the quintiles of urbanicity scores also showed quite obvious dose–response patterns of increase in both outcomes across quintiles of urbanicity, especially for body mass index (Fig. 2).

**Table 4:** Construct validity of three urbanicity scales

Test parameter		Novak et al.	Dahly and Adair	Jones-Smith and Popkin
Per Capita Monthly Income (Baht)				
	Raw Urbanicity Score			
	Coefficient (SE)	191 (13)	158 (16)	114 (7)
	(95 % Confidence interval)	(166, 217)	(126, 189)	(100,129)
	*p-value*	*<0.001*	*<0.001*	*<0.001*
	Standardized Urbanicity Score			
	Coefficient (SE)	134 (9)	110 (11)	136 (9)
	(95 % Confidence interval)	(116, 152)	(88, 132)	(120, 154)
	*p-value*	*<0.001*	*<0.001*	*<0.001*
Body Mass Index (kg/m2)^a^				
	Raw Urbanicity Score			
	Coefficient (SE)	0.064(0.004)	0.063 (0.005)	0.036 (0.003)
	(95 % Confidence interval)	(0.056, 0.073)	(0.053, 0.073)	(0.031, 0.041)
	*p-value*	*<0.001*	*<0.001*	*<0.001*
	Standardized Urbanicity Score			
	Coefficient (SE)	0.045 (0.003)	0.044 (0.004)	0.042 (0.003)
	(95 % Confidence interval)	(0.039, 0.051)	(0.037, 0.051)	(0.036, 0.049)
	*p-value*	*<0.001*	*<0.001*	*<0.001*

^a^Adjusted for age and gender

## Discussion

Validity and reliability of an urbanicity scale is of utmost importance in the development of effective strategies to minimize adverse social and health consequences of increased urbanization [17]. This study evaluated the comparability and robustness of the previously validated urbanicity scales proposed by Dahly and Adair [7], Novak et al. [15], and Jones-Smith and Popkin [14] when utilized in a Thailand context. Results showed that while correlation among the three scales was high, those proposed by Dahly and Adair and Jones-Smith and Popkin were more comparable. As for the properties of the scales, all three scales had good criterion-related validity (as demonstrated by the significant differences in the mean urbanicity scores across the official urban–rural dichotomy and four-category urbanicity classification) and construct validity (as demonstrated by their significant association with the mean per capita monthly income and body mass index). The unidimensionality assumption was, however, attained only for Dahly and Adair’s scale, and the internal consistency was satisfactory only for Jones-Smith and Popkin’s. Overall, Jones-Smith and Popkin’s scale had the highest validity and reliability among the three scales.

This evidence ensures the generalizability of the study’s findings about the association of urbanicity/urbanization with social and health impacts from one area to another in developing countries. However, when the urbanicity level is the main interest, caution is required when comparing different studies since the urbanicity scales used in the studies might not be comparable.

Although the urbanicity scores and existing official urban–rural classifications were highly correlated, the quantitative nature of the formers render their superiority over the latters in facilitating the detection of more delicate patterns of the health and social impacts of urbanicity/urbanization (such as nonlinearity pattern, differential impacts among communities within the same category of urban–rural classification) [43]. Since communities in the same category of the official urban–rural classifications are actually heterogeneous in terms of development level, the qualitative nature of the official urban–rural classifications may obscure nuance, or significant details of urbanicity/urbanization impacts. This issue has already been demonstrated in a number of previous studies [7, 14, 18, 19].

Since the proposed components in these three scales - based on the existing literature - were all associated with urbanicity, unidiemnsionality was therefore assumed for these scales [9, 44–46]. However, when applying these scales to this study’s context, two out of three of the scales did not comply with this assumption. The only scale with unidimensionality actually had relatively low internal consistency, which may cause a misleading conclusion about its dimensionality [47]. In addition, the magnitude of internal consistency and criterion-related validity was also less when compared to the original validation results. However, items with low inter-correlations and/or no unidimensionality can yield an interpretable scale provided that a large proportion of the test variance is attributable to the first principal factor, as is the case for these scales [47].

This study’s variant findings about dimensionality and internal consistency of the urbanicity scales might be due to many possibilities. The most likely explanation is the differences in the data sources, the definition of variables, and their measurement methods used in the scale composition. This study relied solely on existing secondary data on village, sub-district, and even district levels. Definition and scoring procedure of the urbanicity related variables, therefore, had to be made to accommodate the available data. The application of Dahly and Adair’s and Jones-Smith and Popkin’s scales was largely affected by these issues, since a signification modification had been done. One example is that stricter definitions were given for sewage treatment system and bus station; another was relying on data at district level for housing related variables, which resulted in less variability of these two components in the Jones-Smith and Popkin’s scale. Relying on primary data collection may minimize these differences and improve the validity and reliability of the scales; however, this will require a higher budget.

In addition, the differences in the boundary of the study community can also be another explanation, especially for Novak et al’s scale. Based on the information on the average number of population in the community of Novak et al’s scale (8,538 and 3,855 for mean and median, respectively) and this study’s (621 and 508) [15], their community was comparable to this study’s sub-district rather than village. This affected this study’s differential scoring results of many urbanicity components, particularly population size and educational facilities in a locality, which had very low correlation with other urbanicity components and diverged from the main factor in the factor analysis.

Since a significant proportion of data were imputed in this study, inaccurate data and bias from improper imputation might also be another possible explanation. The most concern was for the imputation of some data of the municipal communities by data from the most comparable villages (with possibly lower urbanized level). Some component scores (such as population size and density and paved road density) of these communities might therefore have been underestimated, resulting in lower correlation and failure to achieve unidimensionality among the component scores of the municipal communities. This possibility was examined by reanalyzing the data without these communities in the dataset. The results were however not significantly changed (details not shown).

Lastly, in real world data, the assumption on strict unidimentionality may not be practical [48]. In this case, the “essential” unidimensionality — which may reflect several traits but one that very clearly dominates - may be more appropriate [48]. Since urbanicity and urbanization have multiple determinants and their stage and pattern are heterogeneous in different areas, both among and within countries, this supposition may be relevant [49, 50]. However, due to low communality (<0.20) and the skew of some study variables, this study was unable to examine this issue as a larger sample size is required. This issue needs further investigation.

The Jones-Smith and Popkin’s scale seemed to be slightly superior to the other two scales in terms of internal consistency as well as criterion-related and construct validities. In addition to its higher Pearson’s Spearman’s correlation coefficients and Kappa statistic (Table 3), its scores were well distributed in the stepwise manner according to the official four-category urban classification (Fig. 1). Furthermore, per capita monthly income and body mass index did significantly increase across the quintiles of the urbanicity score (Fig.2c and (f) ). This was not always the case for the other two scales. The Jones-Smith and Popkin’s scale differed from the other two scales in many ways, including its larger number of components and finer gradation in the scoring of transportation, health, and modern market components [14]. In the scoring of these components, size, number, and proximity of the institutions/facilities/services were taken into consideration, resulting in higher variability of the urbanicity component scores. However, it must be weighed against higher requirements for more detailed data that might not be available/exist in certain countries. These observations can be useful for the future development or improvement of urbanicity scales.

Notwithstanding the above defect, all scales worked well in terms of criterion-related validity and construct validity. This was quite consistent with existing evidence on the relationship of urbanicity with health and social parameters, including per capita income and body mass index. For body mass index, this study provides firmer evidence by minimizing the potential confounding effect of age and gender in the analysis of urbanicity level and body mass index relationship. This means that the study’s findings about urbanicity and health and social impacts by using these urbanicity scales can be generalized internationally.

Although the sample size was quite large (537 villages and communities) compared to previous studies (118–270 villages/communities) and represented the whole country, some limitations need mentioning. First, communities in Bangkok, the capital city of Thailand, were not included in this study, since detailed community-specific data were not available. The extent of applicability and robustness of the urbanicity scales when utilizing in highly urbanized areas is still unconfirmed. Second, the validity of Van de Poel et al’s scale (of which the scoring system is based on factor analysis) was not able to be verified in this study due to a lack of relevant data as mentioned previously. Last, since a number of modifications on the original scales had been made in this study, it is still uncertain that some altered validation results are due to these modifications or the properties of the original urbanicity scales. Future studies that rely on primary data collection could make issues resulting from these limitations clearer.

## Conclusions

This study showed that the urbanicity scales proposed by Dahly and Adair [7], Novak et al. [15], and Jones-Smith and Popkin [14] were robust in terms of their degree of agreement with the existing official urban–rural classification (i.e. criterion-related validity) and their coincidence with the phenomena known to differ by urban status (i.e. construct validity) when applying in a country other than their originally developed locations. Their utilities as valid instruments for examining the social and health impacts of urbanicity/urbanization in the international context are therefore insured. However, the comparison of urbanicity levels across different countries must be cautious when different urbanicity scales are used.

## Additional file

Additional file 1:
**Scale scoring algorithms.** (DOCX 60 kb)
