# Supplementary material for: Validation and comparison study of three urbanicity scales in a Thailand context
Source: BMC Public Health. 2016 Jan 14;16:34. doi: 10.1186/s12889-016-2704-y (PMC4712519; doi:10.1186/s12889-016-2704-y)
Supplement: Additional file 1: — Scale scoring algorithms. (DOCX 60 kb) [file 12889_2016_2704_MOESM1_ESM.docx]

**Supplementary file**

**Dahly and Adair's Urbanicity Scale**

| **Component** | **Score Item** | | **Scale Scoring** | **Data Source** | **Remark** |
| --- | --- | --- | --- | --- | --- |
| Population characteristics | Points Size | Density (persons per km^2^) |  | DCD |  |
|  | 1–500 | 1–500 | 1 point |  |  |
|  | 501–1000 | 501–1000 | 2 points |  |  |
|  | 1001–2000 | 1001–2500 | 3 points |  |  |
|  | 2001–4000 | 2501–5000 | 4 points |  |  |
|  | 4001–6000 | 5001–7500 | 5 points |  |  |
|  | 6001–8000 | 7501–10,000 | 6 points |  |  |
|  | 8001–10,000 | 10,001–15,000 | 7 points |  |  |
|  | 10,001–15,000 | 15,001–30,000 | 8 points |  |  |
|  | 15,001–20,000 | 30,001–50,000 | 9 points |  |  |
|  | >20,000 | >50,000 | 10 points |  |  |
| **Communications** | Communication services were available in the locality | | (Adjust to 10) |  | † |
|  | mail service | | 2 points | DCD |  |
|  | telephone service | | 3 points | DCD |  |
|  | cell phone service | | 1 point | DCD |  |
|  | internet service | | 1 point | DCD |  |
|  | cable service | | 1 point | NSO | ¶ |
| **Educational facilities** | Educational services were available in the locality | |  |  |  |
|  | Nursery and/or preschool | | 2 points | DCD | † |
|  | primary intermediate schools | | 2 points | DCD |  |
|  | secondary schools | | 2 points | DCD |  |
|  | vocational training facilities | | 2 points | OVEC |  |
|  | college | | 2 points | OHEC |  |
| **Transportation** | the presence and availability of bus, taxi, and motorcycle service | |  | DCD | † |
|  | continuous service | | 3 points |  |  |
|  | any daily service | | 2 points |  |  |
|  | less than daily service | | 1 point |  |  |
|  | no service | | 0 points |  |  |
|  | paved road density (km road/km2) | |  | DCD |  |
|  | no paved road | | 0 points |  |  |
|  | 0.001–0.500 | | 1 point |  |  |
|  | 0.501–1.000 | | 2 points |  |  |
|  | 1.001–5.000 | | 3 points |  |  |
|  | > 5.000 | | 4 points |  |  |
| **Health services** | Health services available in the locality | |  |  | † |
|  | any hospital | | 3 points | BSAH |  |
|  | private medical clinics | | 2 points | BSAH |  |
|  | pharmacies | | 1 point | BDC |  |
|  | medical clinics | | 1 point | BSAH |  |
|  | nurse and midwifery clinics | | 1 point | BSAH |  |
|  | dental clinics | | 1 point | BSAH |  |
|  | physiotherapy clinic | | 1 point | BSAH |  |
|  | Medical technician clinic | | 1 point | BSAH |  |
| **Markets** | Presence of commercial services | | (Adjusted to 10) |  | † |
|  | fresh markets | | 2 points | BFWS |  |
|  | gas stations | | 2 points | DCD |  |
|  | drug stores | | 1 point (skip) |  |  |
|  | the number of small grocery’ stores | |  | DCD |  |
|  | 0 stores | | 0 point |  |  |
|  | 1–20 stores | | 1 point |  |  |
|  | 21–50 stores | | 2 points |  |  |
|  | 51–100 stores | | 3 points |  |  |
|  | 101–200 stores | | 4 points |  |  |
|  | 200+ stores | | 5 points |  |  |

† modified from the original scale ‡Data at district level ¶Data at sub-district level

**Data source:**

| **Abbreviation** | **Organization** | **Reference** |
| --- | --- | --- |
| BDC | Bureau of Drug Control,Ministry of Public Health, Thailand. | 31 |
| BFWS | Bureau of Food and Water Sanitation, Department of Health, Ministry of Public Health, Thailand. | 25 |
| BSAH | Bureau of Sanatorium and Art of Healing, Department of Health Service Support, Ministry of Public Health**,** Thailand**.** | 23 |
| DCD | Department of Community Development, Ministry of Interior, Thailand. | 21 |
| NSO | National Statistical Office, Ministry of Information Technology and Communication, Thailand. | 22 |
| OHEC | Office of the Higher Education Commission, Ministry of Education, Thailand. | 26 |
| ONREPP | Office of Natural Resources and Environmental Policy and Planning, Ministry of Natural Resources and Environment, Thailand. |  |
| OVEC | Office of the Vocational Education Commission, Ministry of Education, Thailand. | 27 |

**Jones-Smith JC and Popkin’s Urbanicity Scale**

| **Component** | **Score Item** | | | | | **Scale Scoring** | **Data Source** | **Remark** |
| --- | --- | --- | --- | --- | --- | --- | --- | --- |
| Population characteristics | **Population Density** (people per km^2^) | | | | |  | DCD |  |
|  | Log Population Range | | | Population Range | |  |  |  |
|  | 0 | 0.58 | | 1 | 1 | 0 Points |  |  |
|  | 0.58 | 1.16 | | 1 | 3 | 0.5 Points |  |  |
|  | 1.16 | 1.74 | | 3 | 5 | 1 Points |  |  |
|  | 1.74 | 2.32 | | 5 | 10 | 1.5 Points |  |  |
|  | 2.32 | 2.9 | | 10 | 18 | 2 Points |  |  |
|  | 2.9 | 3.48 | | 18 | 32 | 2.5 Points |  |  |
|  | 3.48 | 4.06 | | 32 | 57 | 3 Points |  |  |
|  | 4.06 | 4.64 | | 57 | 103 | 3.5 points |  |  |
|  | 4.64 | 5.22 | | 103 | 184 | 4 points |  |  |
|  | 5.22 | 5.8 | | 184 | 330 | 4.5 points |  |  |
|  | 5.8 | 6.38 | | 330 | 589 | 5 points |  |  |
|  | 6.38 | 6.96 | | 589 | 1053 | 5.5 points |  |  |
|  | 6.96 | 7.54 | | 1053 | 1881 | 6 points |  |  |
|  | 7.54 | 8.12 | | 1881 | 3361 | 6.5 points |  |  |
|  | 8.12 | 8.7 | | 3361 | 6002 | 7 points |  |  |
|  | 8.7 | 9.28 | | 6002 | 10721 | 7.5 points |  |  |
|  | 9.28 | 9.86 | | 10721 | 19148 | 8 points |  |  |
|  | 9.86 | 10.44 | | 19148 | 34200 | 8.5 points |  |  |
|  | 10.44 | 11.02 | | 34200 | 61083 | 9 points |  |  |
|  | 11.02 | 11.6 | | 61083 | 109097 | 9.5 points |  |  |
|  | 11.6 | 12.18 | | 109097 | 194852 | 10 points |  |  |
| **Economic** | • Real daily wage for ordinary male worker | | | | | 10 points | DCD |  |
|  | • Percent of community engaged in non-agriculture | | | | |  | DCD |  |
|  | If daily wage>199 baht (official minimum daily wage in Bangkok in 2007), then econ score = 2 * (proportion not in agriculture))/20 | | | | |  |  |  |
|  | If male wage<199 baht, then econ score = 1*(proportion not in agriculture))/20 | | | | |  |  |  |
| **Traditional**  **markets** | **Types of goods/markets available:** | | | | |  |  |  |
|  | Presence of fresh market (everyday) | | | | |  | BFWS | †¶ |
|  | Yes | | | | | 10 points |  |  |
|  | No | | | | | 0 point |  |  |
| **Supermarket and food**  **vendors** | **supermarkets** | | | | |  |  |  |
|  | supermarkets are within 30 min bus ride | | | | |  | TYP |  |
|  | 0 | | | | | 0 point |  |  |
|  | 1 | | | | | 1 point |  |  |
|  | 2+ | | | | | 2 points |  |  |
|  |  | | | | |  |  |  |
|  | **Food vendors** | | | | |  |  |  |
|  | number of food vendors in locality | | | | |  | DCD |  |
|  | 0 | | | | | 0 point |  |  |
|  | 1 to 2 | | | | | 1 point |  |  |
|  | 3 to 4 | | | | | 2 points |  |  |
|  | 5 to 7 | | | | | 3 points |  |  |
|  | 8 to 10 | | | | | 4 points |  |  |
|  | 11 to 20 | | | | | 5 points |  |  |
|  | 21 to 50 | | | | | 6 points |  |  |
|  | 51 to 100 | | | | | 7 points |  |  |
|  | < 100 | | | | | 8 points |  |  |
| Education | Average highest education attainment among adults | | | | | 10 points | DCD | † |
|  | Take actual community average and multiply it by 10/5 to scale the whole range to 10. Round down to 10 if >5. | | | | |  |  |  |
|  | 0-6: 0=none, 1=graduated primary school, 2=lower middle school, 3=upper middle school, 4=middle, technical or vocational, 5=3 or 4 year college degree | | | | |  |  |  |
| **Diversity** | - **Community variance in adult education level** | | | | |  | DCD |  |
|  | Education: Created a variable that is the variance in education for each community each year. | | | | |  |  |  |
|  | Education Variance (comm_var) | | | | | Points |  |  |
|  | 0-0.50 | | | | | 0 point |  |  |
|  | 0.51-0.75 | | | | | 1 points |  |  |
|  | 0.76-1.00 | | | | | 2 points |  |  |
|  | 1.01-1.25 | | | | | 3 points |  |  |
|  | 1.26-1.50 | | | | | 4 points |  |  |
|  | 1.51-1.75 | | | | | 4.5 points |  |  |
|  | 1.76-2.00 | | | | | 5 points |  |  |
|  | 2.01-2.25 | | | | | 6 points |  |  |
|  | 2.26-2.50 | | | | | 7 points |  |  |
|  | 2.51-2.75 | | | | | 8 points |  |  |
|  | 2.76-3.00 | | | | | 9 points |  |  |
|  | >3.00 | | | | | 10 points |  |  |
|  | - **Community variance in income level** | | | | |  | NSO |  |
|  | Income Diversity: Created a variable that is the variance in income for each community in each year (Scale based on log scale for huge range in numbers). | | | | |  |  |  |
|  |  | | | | |  |  |  |
|  |  | | | | |  |  |  |
|  |  | | | | |  |  |  |
|  |  | | | | |  |  |  |
|  | Income Variation | | | | |  |  |  |
|  | From | | To | | | Points |  |  |
|  | >0 | | 20 | | | 1 points |  |  |
|  | >20 | | 90 | | | 2 points |  |  |
|  | >90 | | 400 | | | 3 points |  |  |
|  | >400 | | 1800 | | | 4 points |  |  |
|  | >1800 | | 8100 | | | 5 points |  |  |
|  | >8100 | | 36300 | | | 6 points |  |  |
|  | >36300 | | 162750 | | | 7 points |  |  |
|  | >162750 | | 729400 | | | 8 points |  |  |
|  | >729400 | | 3269000 | | | 9 points |  |  |
|  | >3269000 | |  | | | 10 points |  |  |
|  | For total score, take mean of Education Diversity and Income Diversity scores | | | | |  |  |  |
| **Health** | Type of healthcare facilities available in locality or nearby | | | | |  |  |  |
|  | Village clinic or Private Clinic | | | | | 1 point | BSAH |  |
|  | Township hospital | | | | | 2 points | BSAH |  |
|  | County hospital | | | | | 3 points | BSAH |  |
|  | City hospital | | | | | 4 points | BSAH |  |
|  | An additional 1 point for pharmacy/drug store in community only (i.e. not nearby) | | | | | 1 point | BDC |  |
|  | **Then** | | | | |  |  |  |
|  | Let total possible from above equal 8 points only | | | | |  |  |  |
|  | **Then** | | | | |  |  |  |
|  | Adjust up if they have more than one clinic: | | | | |  | BSAH |  |
|  | multiply their score by 1.25 so that their total can equal 10 | | | | |  |  |  |
| **Transportation** | **Type of road:** | | | | |  | DCD |  |
|  | dirt | | | | | 0 point |  |  |
|  | stone/gravel/mixed | | | | | 1 point |  |  |
|  | paved | | | | | 2 points |  |  |
|  | **Bus station (Long distance bus station):** | | | | |  | BPT | †¶ |
|  | in sub-district | | | | | 2 points |  |  |
|  | no close bus | | | | | 0 point |  |  |
|  | **Train station:** | | | | |  | WiKi-1 |  |
|  | in community | | | | | 2 points |  |  |
|  | close, but not in community | | | | | 1 points |  |  |
|  | no close train | | | | | 0 points |  |  |
|  | Scaled to 10 points = (road + bus + train)*1.6666 | | | | |  |  |  |
|  |  | | | | |  |  |  |
| **Housing** | **In house Electricity** | | | | | 2.5 points | DCD |  |
|  | points = percent of community with electricity/10 | | | | |  |  |  |
|  | **In house tap water** | | | | | 2.5 points | DCD |  |
|  | point = percent of community with indoor piped water/10 | | | | |  |  |  |
|  | **In house flush toilet** | | | | | 2.5 points | NSO | ‡ |
|  | point = percent of community with flush toilet/10 | | | | |  |  |  |
|  | **Cooking with gas** | | | | | 2.5 points | NSO | ‡ |
|  | point=percent of community that cooks with gas/10 | | | | |  |  |  |
|  | For score take the mean of electricity, water, toilet and gas | | | | |  |  |  |
| **Sanitation** | **Treated water** | | | | |  | ONREPP | † |
|  | Presence of well equipped sewage treatment system in locality | | | | |  |  |  |
|  | Yes | | | | | 10 points |  |  |
|  | No | | | | | 0 points |  |  |
| **Communications** |  | | | | | 10 points |  | † |
|  | Television | | | | |  |  |  |
|  | Percent of households with color TV/10 | | | | |  | NSO | ‡ |
|  | Computer | | | | |  |  |  |
|  | Percent of households with computer/10 | | | | |  | DCD |  |
|  | Cellphone | | | | |  |  |  |
|  | Percent of households with cellphone/10 | | | | |  | DCD |  |
|  | Take mean of above 3 numbers and divide by 1.66666 (or 10/6). | | | | |  |  |  |
|  | Then | | | | |  |  |  |
|  | Add 1 point for each of the following: | | | | |  |  |  |
|  | Cinema in neighborhood | | | | |  | WiKi-2 |  |
|  | Postal service available | | | | |  | DCD |  |
|  | Telephone service available | | | | |  | DCD |  |
| **Social Services** | Childcare center | | | | |  | DCD | † |
|  | A child care center for <3year olds; | | | | | 2.5 points |  |  |
|  | If childcare center is not in village/neighborhood, but is in a neighboring village less than 25 km away, give only 1.25 points for this. | | | | | 1.25 points |  |  |
|  | free medical insurance in community | | | | | 2.5 points | NHSO |  |
|  | community vocational training center | | | | | 2.5 points | DCD |  |
|  | guardian center for various kinds of disable persons in sub-district | | | | | 2.5 points | OSCNSWP | ¶ |

† modified from the original scale ‡Data at district level ¶Data at sub-district level

**Data source:**

| **Abbreviation** | **Organization** | **Reference** |
| --- | --- | --- |
| BDC | Bureau of Drug Control,Ministry of Public Health, Thailand. | 31 |
| BFWS | Bureau of Food and Water Sanitation, Department of Health, Ministry of Public Health, Thailand. | 25 |
| BPT | Bureau of Passenger Transport, Department of Land Transportation, Ministry of Transport**,** Thailand**.** | 29 |
| BSAH | Bureau of Sanatorium and Art of Healing, Department of Health Service Support, Ministry of Public Health**,** Thailand**.** | 23 |
| DCD | Department of Community Development, Ministry of Interior, Thailand. | 21 |
| NHSO | National Health Security Office of Thailand. |  |
| NSO | National Statistical Office, Ministry of Information Technology and Communication, Thailand. | 22 |
| ONREPP | Office of Natural Resources and Environmental Policy and Planning, Ministry of Natural Resources and Environment, Thailand. | 28 |
| OSCNSWP | Office of the Support Committee for the National Social Welfare Provision, Department of Social Development and Welfare, Ministry of Social Development and Human Security, Thailand. | 36 |
| TYP | Thailand Yellow Pages, Teleinfo Media Public Company Limited, Thailand. | 33 |
| WiKi-1 | Wikipedia ( List of railway stations by the State Railway of Thailand). | 24 |
| WiKi-2 | Wikipedia (List of Movie Theatres in Thailand). | 30 |

**Novak et al' Urbanicity Scale**

| **Component** | **Score Item** | **Scale Scoring** | **Data Source** | **Remark** |
| --- | --- | --- | --- | --- |
| Population size | Approximate number of people (including children) living in the locality |  | DCD |  |
|  | 1–500 | 1 point |  |  |
|  | 501–1,000 | 2 points |  |  |
|  | 1,001–2,000 | 3 points |  |  |
|  | 2,001–4,000 | 4 points |  |  |
|  | 4,001–6,000 | 5 points |  |  |
|  | 6,001–8,000 | 6 points |  |  |
|  | 8,001–10,000 | 7 points |  |  |
|  | 10,001–15,000 | 8 points |  |  |
|  | 15,001–20,000 | 9 points |  |  |
|  | >20,000 | 10 points |  |  |
| Economic activity | Proportion of the population involved in agriculture (primary occupation)* | 10 points – 10 x proportion of population involved in agriculture* | DCD |  |
| Built environment | **Types of roads in locality** |  | DCD |  |
|  | Paved roads | 2 points |  |  |
|  | Unpaved roads for motor traffic | 1 point |  |  |
|  | Non-motorised roads | 0 points |  |  |
|  | **Sewage services** |  |  |  |
|  | Sewage system in locality | 2 points | ONREPP |  |
|  | Proportion of households with a flush toilet | 2 points x proportion of households with a flush toilet | NSO | ‡ |
|  | **Electricity services** |  |  |  |
|  | Electricity in locality | 2 points | DCD |  |
|  | Proportion of households with electricity | 2 points x proportion of households with electricity | DCD |  |
| Communication services | Communication services in locality |  |  |  |
|  | Proportion of households with television | 2 points | NSO | ‡ |
|  | Proportion of households with mobile phone | 2 points | DCD |  |
|  | Movie theatre | 2 points | WiKi-2 | ‡ |
|  | Public internet | 2 points | DCD |  |
|  | Public telephone | 2 points | DCD |  |
| Education facilities | Educational facilities in locality |  |  |  |
|  | Nursery and/or preschool | 2 points | DCD |  |
|  | Primary school | 2 points | DCD |  |
|  | Secondary school | 2 points | DCD |  |
|  | University | 2 points | OHEC |  |
|  | Average education of adult female in the locality (year) | Average number of years of education/6‘ | DCD | † |
|  |  |  |  |  |
|  |  |  |  |  |
| Health services | Health facilities in locality |  |  |  |
|  | Hospital (public or private) | 2 points | BSAH |  |
|  | Health centre (public or private) | 2 points | BSAH |  |
|  | Dispensary/pharmacy | 2 points |  |  |
|  | Health workers available in locality |  |  |  |
|  | Midwife | 2 points | BSAH |  |
|  | Health worker | 2 points | PHCD |  |
| Diversity | Variance in per capita monthly income |  | NSO |  |
|  | Decile 10 | 5 points |  |  |
|  | Decile 9 | 4.5 points |  |  |
|  | Decile 8 | 4 points |  |  |
|  | Decile 7 | 3.5 points |  |  |
|  | Decile 6 | 3 points |  |  |
|  | Decile 5 | 2.5 points |  |  |
|  | Decile 4 | 2 points |  |  |
|  | Decile 3 | 1.5 points |  |  |
|  | Decile 2 | 1 point |  |  |
|  | Decile 1 | 0.5 points |  |  |
|  | Variance in adult female’s education |  | DCD |  |
|  | Decile 10 | 5 points |  |  |
|  | Decile 9 | 4.5 points |  |  |
|  | Decile 8 | 4 points |  |  |
|  | Decile 7 | 3.5 points |  |  |
|  | Decile 6 | 3 points |  |  |
|  | Decile 5 | 2.5 points |  |  |
|  | Decile 4 | 2 points |  |  |
|  | Decile 3 | 1.5 points |  |  |
|  | Decile 2 | 1 point |  |  |
|  | Decile 1 | 0.5 points |  |  |

† modified from the original scale ‡Data at district level

**Data source:**

| **Abbreviation** | **Organization** | **Reference** |
| --- | --- | --- |
| BSAH | Bureau of Sanatorium and Art of Healing, Department of Health Service Support, Ministry of Public Health**,** Thailand**.** | 23 |
| DCD | Department of Community Development, Ministry of Interior, Thailand. | 21 |
| NSO | National Statistical Office, Ministry of Information Technology and Communication, Thailand. | 22 |
| OHEC | Office of the Higher Education Commission, Ministry of Education, Thailand. | 26 |
| ONREPP | Office of Natural Resources and Environmental Policy and Planning, Ministry of Natural Resources and Environment, Thailand. | 28 |
| PHCD | Primary Health Care Division, Department of Health Service Support, Ministry of Public Health, Thailand. | 32 |
| WiKi-2 | Wikipedia (List of Movie Theatres in Thailand). | 30 |
